# Restricted distribution and population genetic structure in the rare Korean endemic plant *Veronica pusanensis*: implications for conservation

**DOI:** 10.3389/fpls.2026.1861445

**Published:** 2026-07-29

**Authors:** Kyu Tae Park, Young-Ho Kim, Sang-Jun Kim, KyoungSu Choi

**Affiliations:** 1 Department of Biology, College of Natural Science, Kyungpook National University, Daegu, Republic of Korea; 2 Bio-Resource Research Center, Kyungpook National University, Daegu, Republic of Korea; 3 School of Life Sciences, BK21 FOUR KNU Creative BioResearch Group, Kyungpook National University, Daegu, Republic of Korea; 4 Forest Biodiversity Conservation Research Division, Korea National Arboretum, Pocheon, Republic of Korea

**Keywords:** 3RAD-seq, conservation genomics, critically endangered (CR), endemic plant, genetic diversity, population structure, *Veronica pusanensis*

## Abstract

**Introduction:**

*Veronica pusanensis* is a critically endangered plant endemic to Korea and is restricted to a single wild population in Busan. Understanding its genetic diversity, population structure, and effective population size is essential for conservation genomics and for evaluating the representativeness of ex situ collections.

**Methods:**

We applied triple-digest restriction site-associated DNA sequencing (3RAD-seq) to compare genetic diversity and population structure between the wild population and four ex situ populations maintained in botanical gardens, identifying 43,885 single-nucleotide polymorphisms (SNPs) across 95 individuals. Clone correction based on the full SNP dataset reduced the dataset to 59 representative multilocus genotypes.

**Results:**

The wild population (GJ) showed moderate genetic diversity compared with other Korean endemic species, and positive inbreeding coefficients were observed in all populations. Population structure analyses based on the clone-corrected and LD-pruned 5,539-SNP dataset revealed genetic differentiation among populations. Principal component analysis showed that GJ and HM clustered closely, whereas GC, HT, and YM showed varying degrees of differentiation. ADMIXTURE analysis identified K = 3 as the best-supported value based on the lowest cross-validation error, with GJ and HM sharing a common ancestry component, YM showing a distinct ancestry pattern, and GC and HT exhibiting mixed ancestry. Pairwise fixation index (*F^ST^*) values ranged from −0.069 to 0.126. Finite effective population size estimates were obtained only for GJ and HM, whereas finite estimates could not be obtained for GC, HT, and YM, likely reflecting the small number of independent individuals remaining after clone correction.

**Discussion:**

Genetic differences among ex situ populations may reflect differences in source material, propagation history, and clonal redundancy. These findings suggest that ex situ groups differ in their ability to represent the genetic composition of the wild population. Conservation of *V. pusanensis* should prioritize in situ habitat protection, the maintenance of genetically distinct lineages in ex situ collections, and improved documentation of source materials and propagation history.

## 1 Introduction

Biodiversity, encompassing the variability of genes, species, and ecosystems, is fundamental to the stability and functioning of natural systems. However, global biodiversity is currently experiencing unprecedented declines due to anthropogenic pressures such as habitat destruction, climate change, pollution, and overexploitation (Díaz et al., 2019; Keck et al., 2025). These threats are particularly severe for endemic and geographically restricted species, which often possess limited population sizes and reduced genetic variability, making them more vulnerable to extinction (Isik, 2011; Glassock et al., 2021; Su et al., 2025).

Traditionally, conservation efforts have largely relied on field-based surveys and ecological observations. However, recent advances in molecular approaches have enabled a deeper understanding of genetic diversity and population structure, which are now recognized as essential components for developing effective conservation strategies (Allendorf et al., 2010; Shafer et al., 2015; Supple and Shapiro, 2018). In particular, next-generation sequencing (NGS) technologies have revolutionized population genetic studies, enabling high-resolution analyses of genetic variation. Reduced-representation sequencing approaches, such as genotyping-by-sequencing (GBS) and restriction site-associated DNA sequencing (RAD-seq), are now widely applied in population studies (Baird et al., 2008; Elshire et al., 2011; Gugger et al., 2021; Xiang et al., 2025).

For narrowly distributed endemic species, genomic data are particularly valuable because they can reveal levels of genetic diversity, population differentiation, and historical demographic change that are not readily detectable from ecological surveys alone. Such information is essential for identifying conservation units and establishing evidence-based management strategies (Stojanova et al., 2020; Cai et al., 2021; Zhao et al., 2023; Gong et al. 2025).

The genus *Veronica* is the largest genus in Plantaginaceae, comprising approximately 450 species worldwide (Albach et al., 2005; Albach and Meudt, 2010). Chromosome number and ploidy level vary among *Veronica* species and species groups, and polyploidy or intraspecific ploidy variation has been reported in several lineages, including species in subgen. *Pseudolysimachium* such as *V. spicata* and *V. longifolia* (Albach and Daubert, 2023). In Korea, 17 species have been reported, including four endemic taxa restricted to the Korean Peninsula: *V. nakaiana* Ohwi, *V. kiusiana* var. *diamantica* (Nakai) T. Yamaz., *V. pyrethina* Nakai, and *V. pusanensis* Y. Lee (Chung et al., 2017). Among these endemic species, *V. pusanensis* belongs to subgen. *Pseudolysimachium* and has recently been reported as diploid, with 2n = 34 chromosomes (Park et al., 2025). It is a perennial herb characterized by stems that grow obliquely rather than erect, opposite leaves that are thick and serrate, and dense coverings of fine white hairs on both leaves and stems. Its flowers bloom from July to August and are borne in dense terminal racemes (Jang and Noh, 2020). The species is currently classified as Critically Endangered (CR) in the Korean Red List of vascular plants (Korea National Arboretum, 2021). Its natural distribution in Busan, South Korea, is restricted to a single known locality. Given its rarity and highly restricted distribution, *V. pusanensis* requires urgent conservation attention. Previous studies (Lee et al., 2021; Park et al., 2025) have focused mainly on propagation and horticultural management to support *ex situ* conservation and cultivation. However, species-specific ecological information directly relevant to population persistence, such as pollination biology, seed dispersal distance and direction, and natural seedling recruitment, remains limited. Therefore, genome-wide genetic data are essential for evaluating whether existing wild and *ex situ* populations retain sufficient genetic diversity for long-term conservation.

Therefore, the objectives of this study were to assess the genetic diversity of *V. pusanensis* using a triple-digest variant of RAD-seq (3RAD-seq) and to compare the genetic composition of the single natural population with that of ex situ–propagated individuals maintained in four botanical gardens. This study further aimed to evaluate whether *ex situ* populations have retained the genetic diversity present in the wild population (GJ) and to provide a genomic basis for the conservation of this critically endangered endemic species.

## 2 Materials and methods

### 2.1 Plant material and DNA extraction

**Figure 1:**
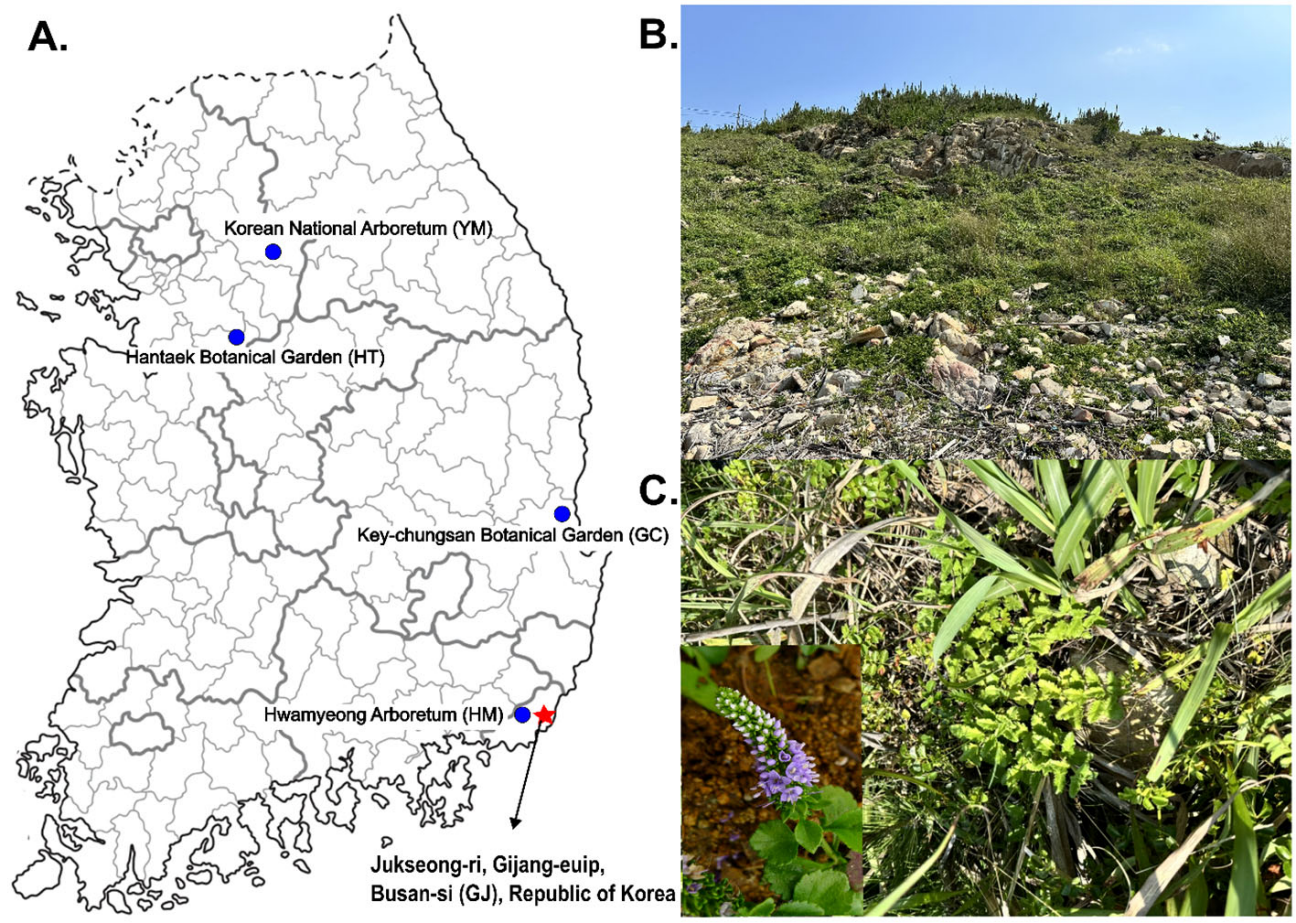
Distribution, habit, and morphological characteristics of *Veronica pusanensis*. **(A)** Sampling locations of the wild population in Busan (red star) and four *ex situ* collections (blue circles) in the Republic of Korea; **(B)** natural habitat of the wild population; **(C)** Morphological characteristics of the leaves and inflorescence.

A total of 95 individuals were sampled from one wild population and four ex situ–propagated groups (**Figure 1**, **Table 1**). For the wild GJ population, leaf samples were collected from spatially separated plants where possible to reduce the likelihood of repeatedly sampling the same genet. For the *ex situ* groups, leaf samples were collected from propagated plants available at each institution at the time of sampling. Fresh young leaves were collected for population genetic analysis and stored at -80 °C until use. Genomic DNA was extracted from leaf tissue using the DNeasy Plant Mini Kit (Qiagen Corporation, Valencia, CA, USA). DNA quality and quantity were assessed by agarose gel electrophoresis.

**Table 1:** Habitat characteristics of *Veronica pusanensis* populations.

Type of samples	Population label	Location	Longitude (E)	Latitude (N)	Initial *N*	Clone-corrected N
Wild population	GJ	Jukseong-ri, Gijang-eup, Busan-si, South Korea	129°14ʹ40ʺ	35°13ʹ52ʺ	20	20
Propagated individuals	GC	Key-Chungsan Botanical Garden, 50, Cheongha-ro, Cheongha-myeon, Pohang-si, South Korea	129°19ʹ57ʺ	36°11ʹ44ʺ	19	7
	HM	Hwamyeong Arboretum, 299, Sanseong-ro, Busan-si, South Korea	129°02ʹ34ʺ	35°15ʹ03ʺ	19	19
	HT	Hantaek Botanical Garden, Hantaek-ro, Cheoin-gu, Yongin-si, South Korea	127°24ʹ23ʺ	37°05ʹ40ʺ	28	5
	YM	Korea National Arboretum, 21-4, Dudam-gil, Yongmum-myeon, Yangpyeong-gun, Gyeonggi-do, South Korea	127°35ʹ52ʺ	37°28ʹ45ʺ	9	8

### 2.2 Draft genome assembly for SNP discovery

A draft genome of *V. pusanensis* was generated from a representative individual collected from the natural GJ population (Busan, South Korea) using PacBio HiFi long-read sequencing data and hifiasm v.0.25.0-r726 with default parameters (Cheng et al., 2021). The resulting draft assembly was used for read alignment and single-nucleotide polymorphism (SNP) calling in downstream analyses. Genome size and sequence characteristics were estimated using GenomeScope 2.0 (Ranallo-Benavidez et al., 2020) based on the *k*-mer distribution of sequencing reads. The *k*-mer histogram was generated with *k* = 21, and the resulting profile was used to infer genome size, heterozygosity, and the proportion of unique and repetitive sequences. The assembly completeness was assessed using BUSCO with the embryophyta_odb 10 dataset (Waterhouse et al., 2018).

### 2.3 3RAD library preparation and sequencing

For 3RAD library preparation, genomic DNA was digested with the restriction enzymes XbaI, NheI, and EcoRI, followed by adapter ligation using a modified version of a previously published protocol (Elshire et al., 2011). After ligation, the samples were pooled, purified with HiAccuBead (AccuGene, Korea) and size-selected prior to PCR amplification. The final 3RAD libraries were sequenced on an Illumina NovaSeq X platform (Illumina Inc., San Diego, CA, USA).

### 2.4 Read preprocessing, alignment, and SNP calling

Raw reads were demultiplexed based on barcode sequences. Adapter trimming was performed using cutadapt v.1.8.3 (Martin, 2011), and quality filtering was conducted using Trimmomatic v.0.39 (Bolger et al., 2014) with the following parameters: 1) sliding window size = 4, mean quality ≥ 15, 2) LEADING and TRAILING ≥ 3, 3) minimum read length ≥ 36 bp. Filtered reads were aligned to the *V. pusanensis* draft genome assembly using BWA-MEM2 (Li and Durbin, 2009) with default parameters. Alignments were converted to BAM format for downstream analysis. Variants were called from the aligned BAM files using DeepVariant v.1.10 (Yun et al., 2021), and SNP loci were integrated across samples to construct a combined SNP matrix.

### 2.5 SNP filtering

To obtain a high-confidence SNP dataset, only biallelic SNPs were retained. Additional filtering criteria included a minimum read depth of ≥ 3, a minor allele frequency (MAF) of > 0.05, and missing data of < 30%, resulting in 43, 885 SNPs across 95 individuals.

Potential clonal or near-clonal individuals were identified using the 43, 885-SNP dataset before LD pruning. Pairwise genetic similarity was calculated within each population as the proportion of identical genotypes among loci with non-missing data in both individuals. Similarity thresholds ranging from 80.0% to 99.5% were examined. The numbers of inferred clonal groups and individuals removed remained stable across a broad threshold range, approximately 85–94%. A threshold of 94% was selected as the most stringent value within this stable range (**Supplementary Figure 1**).

One representative individual was retained from each clone group, with preference given to individuals with lower genotype missingness. Clone correction reduced the dataset from 95 to 59 individuals, comprising GJ (20), GC (7), HM (19), HT (5), and YM (8). In total, 36 individuals were removed following clone correction (**Table 1**, **Supplementary Table 1**).

Linkage disequilibrium pruning was subsequently performed on the clone-corrected dataset using PLINK v1.9 with the parameter --indep-pairwise 50 5 0.2 (Purcell et al., 2007), resulting in 5, 539 SNPs. The clone-corrected and LD-pruned dataset was used for PCA and ADMIXTURE analyses. The clone-corrected dataset before LD pruning was used for genetic diversity analyses. Pairwise FST and contemporary effective population size were estimated as described in the corresponding sections.

The final clone-corrected and LD-pruned 5, 539-SNP dataset and the corresponding population assignment file are provided as **Supplementary Data S1**, **S2**, respectively. The LD-unpruned dataset containing 43, 885 filtered SNPs across the original 95 individuals is provided as **Supplementary Data S3**. Information on clone-group membership, retained representatives, and removed individuals is provided in **Supplementary Table 1**.

### 2.6 Analysis of genetic diversity and differentiation

Genetic diversity within populations was evaluated using the clone-corrected filtered SNP dataset before LD pruning. The observed heterozygosity (*H*o), expected heterozygosity (*H*e), and inbreeding coefficient (*F*_IS_) were calculated in R using the vcfR, adegenet, and hierfstat packages. Genetic differentiation among populations was assessed using pairwise *F_ST_* values estimated from the clone-corrected and LD-pruned 5, 539- SNP dataset with VCFtools based on the Weir and Cockerham method.

To explore demographic history in the wild population, a folded site-frequency spectrum was generated for GJ and analyzed using dadi (Gutenkunst et al., 2009). Constant-size and two-epoch demographic models were compared across multiple projection settings.

### 2.7 Genetic structure analysis

Population structure was inferred using ADMIXTURE (Alexander et al., 2009) based on the clone-corrected and LD-pruned 5, 539-SNP dataset. Prior to analysis, LD-pruning was performed in PLINK v.1.9 (Purcell et al., 2007) using the –indep-pairwise 50 5 0.2 option. The resulting pruned dataset was used for ADMIXTURE analysis, and ancestry proportions were estimated for *K* values ranging from 1 to 10. The optimal *K* value was determined based on the lowest cross-validation (CV) error.

### 2.8 Principal component analysis

Principal component analysis (PCA) was conducted to examine genetic relationships among individuals using the clone-corrected and LD-pruned 5, 539-SNP dataset. The filtered VCF file was converted into PLINK binary format using PLINK v.1.9 (Purcell et al., 2007), and PCA was performed using the –pca 10 option. The proportion of variance explained by each principal component was calculated from eigenvalues generated by PLINK. PCA results were visualized in Python using matplotlib, and the first four principal components were examined.

### 2.9 Contemporary effective population size estimation

Contemporary effective population size (*N*e) was estimated separately for each population using NeEstimator v.2.1 (Do et al., 2014) based on the LD-method. The clone-corrected and LD-pruned 5, 539-SNP dataset was divided by population, and genotype data were converted from PLINK format to Genepop format for input into NeEstimator. Estimates of *N*e and associated confidence intervals were obtained for each population.

## 3 Results

### 3.1 Draft genome analysis

To generate a draft genome assembly for *V. pusanensis*, a SMRTbell library was sequenced on the PacBio Revio platform using HiFi reads. Sequencing produced 49, 982, 932, 949 bp from 6, 565, 969 HiFi reads, with a read N50 of 8, 405 bp, an average read length of 7, 612 bp, and an average read quality of Q44. The draft genome was assembled using hifiasm, resulting in a draft assembly of 737, 053, 019 bp distributed across 909 contigs. The longest contig measured 53, 000, 205 bp, with a contig N50 of 29, 875, 161 bp and a GC content of 37.15% (**Table 2**).

**Table 2:** Summary statistics of the draft genome of *Veronica pusanensis*.

Features	Statistics
Number of contigs	909
Total size	737, 053, 019 bp
Longest size	53, 000, 205 bp
N50 length	29, 875, 161 bp
GC content	37.14%
BUSCO analysis	
Complete BUSCO	1, 541 (95.5%)
Single copy BUSCO	1, 209 (74.9%)
Duplicated BUSCO	332 (20.6%)
Fragmented BUSCO	17 (1.1%)
Missing BUSCO	56 (3.5%)

N50, contig length such that 50% of the genome assembly is contained in contigs of that length or longer; BUSCO, Benchmarking Universal Single-Copy Orthologs.

GenomeScope analysis estimated the genome size of *V. pusanensis* to be approximately 515.36 Mb, with a heterozygosity of 2.28% (**Supplementary Figure 2**). Assembly completeness, assessed using the embryophyte_odb 10 dataset, identified 95.5% complete BUSCOs, including 74.9% single-copy and 20.6% duplicated BUSCOs, while 1.1% were fragmented and 3.5% were missing (**Table 2**). Given that *V. pusanensis* has recently been reported as diploid with 2n = 34 chromosomes (Park et al., 2025), the duplicated BUSCOs were not considered evidence of polyploidy. Accordingly, this assembly was treated as a draft genomic resource for read mapping and SNP discovery rather than as a fully curated reference genome.

### 3.2 3RAD sequencing

A total of 164 Gb of 3RAD sequencing data was generated from 95 individuals. The average GC content was 38.1%, and the Q30 value was 97.5%. After demultiplexing, 97.59% of reads were retained, and subsequent trimming yielded 1, 055, 247, 522 reads. These reads were mapped to the draft genome assembly with an average mapping rate of 98.79%.

Variant calling initially identified 490, 147 SNP loci. After applying the filtering criteria, 43, 885 high-quality SNPs were retained across 95 individuals. Putative clonal or near-clonal genotypes were then identified using the full 43, 885-SNP dataset before LD pruning. Clone correction reduced the dataset from 95 sampled individuals to 59 representative multilocus genotypes. Linkage disequilibrium pruning was subsequently performed on the clone-corrected dataset, resulting in 5, 539 SNPs for downstream PCA, ADMIXTURE, pairwise FST, and contemporary Ne analyses.

### 3.3 Genetic diversity

Genetic diversity varied among populations based on the clone-corrected filtered SNP dataset before LD pruning (**Table 3**). The number of polymorphic SNPs ranged from 30, 998 in YM to 36, 733 in GJ. Observed heterozygosity (*Ho*) ranged from 0.1750 in GJ to 0.2346 in GC, whereas expected heterozygosity (*He*) ranged from 0.2748 in HM to 0.3430 in HT. The inbreeding coefficient (*F_IS_*) was positive in all populations and ranged from 0.2121 in GC to 0.3760 in GJ.

**Table 3:** Population genetic statistics of *Veronica pusanensis*.

Population	*N*	Polymorphic SNPs	*H*_O_	*H*e	*F_IS_*
GJ	20	36, 733	0.1750	0.2926	0.3760
GC	7	35, 429	0.2346	0.3181	0.2121
HM	19	34, 543	0.1942	0.2748	0.2761
HT	5	32, 633	0.1776	0.3430	0.3738
YM	8	30, 998	0.1787	0.2820	0.3050

*N*, sample size; *H*o, observed heterozygosity; *H*e, expected heterozygosity; *F*_IS_, inbreeding coefficient within population.

### 3.4 Population structure analysis

**Figure 2:**
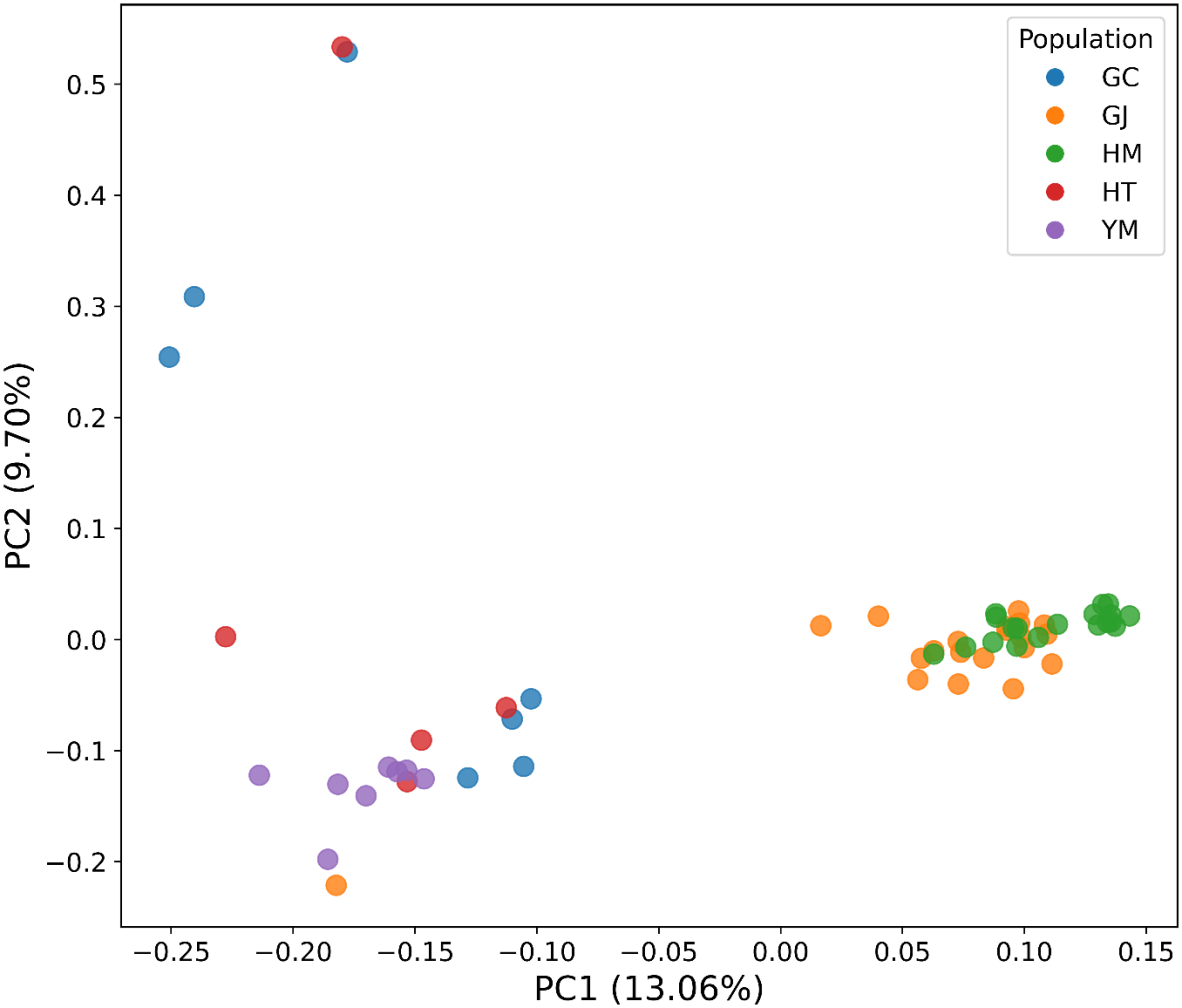
Principal component analysis (PCA) of *Veronica pusanensis* based on the clone-corrected and LD-pruned 5, 539-SNP dataset. The plot illustrates genetic relationships among individuals along PC1 and PC2.

Population structure was investigated using PCA and ADMIXTURE based on the clone-corrected and LD-pruned 5, 539-SNP dataset. PCA revealed clear genetic differentiation among populations, with the first two principal components explaining 13.06% (PC1) and 9.70% (PC2) of the total genetic variation (**Figure 2**). Individuals from HT were separated from the GJ-HM cluster along PC1 and partially overlapped with GC and YM individuals. In contrast, individuals from GJ and HM clustered closely, reflecting a high degree of genetic similarity. The GC population showed a broad distribution, with some individuals separated along PC2, whereas YM was mainly distributed on the negative side of PC1 and PC2. An additional PCA plot based on PC3 and PC4 was generated to further examine variation among individuals. PC3 and PC4 explained 6.89% and 5.88% of the total variation, respectively, but the PC3 and PC4 plot did not reveal additional major population structure beyond that observed in the PC1 and PC2 plot (**Supplementary Figure 3**).

**Figure 3:**
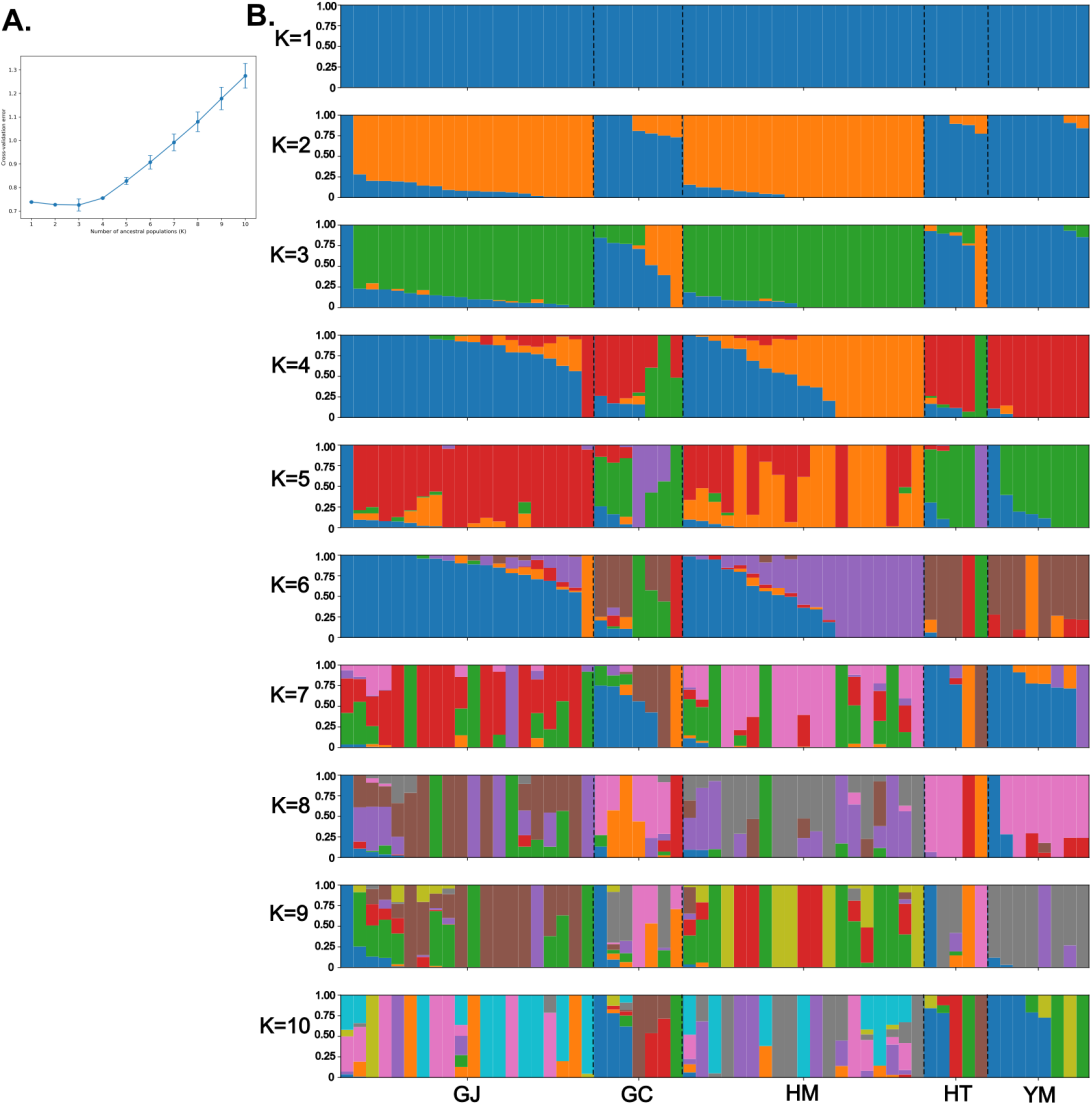
Population genetic structure of *Veronica pusanensis*. **(A)** Cross-validation error from ADMIXTURE analyses, showing that the lowest mean CV error was observed at K = 3. Error bars indicate variation among independent runs. **(B)** ADMIXTURE bar plots for K = 1–10 based on the clone-corrected and LD-pruned 5, 539-SNP dataset. Each vertical bar represents an individual, and the colors indicate the estimated proportion of ancestry assigned to each genetic cluster. Individuals are grouped by population along the x-axis.

ADMIXTURE analysis identified K = 3 as the best-supported value based on the lowest CV error (**Figure 3A**). At this level, GJ and HM shared a common ancestry component, whereas YM was predominantly assigned to a different component. GC and HT exhibited mixed ancestry patterns (**Figure 3B**). These results were generally consistent with the PCA analysis.

### 3.5 Genetic differentiation among populations

**Figure 4:**
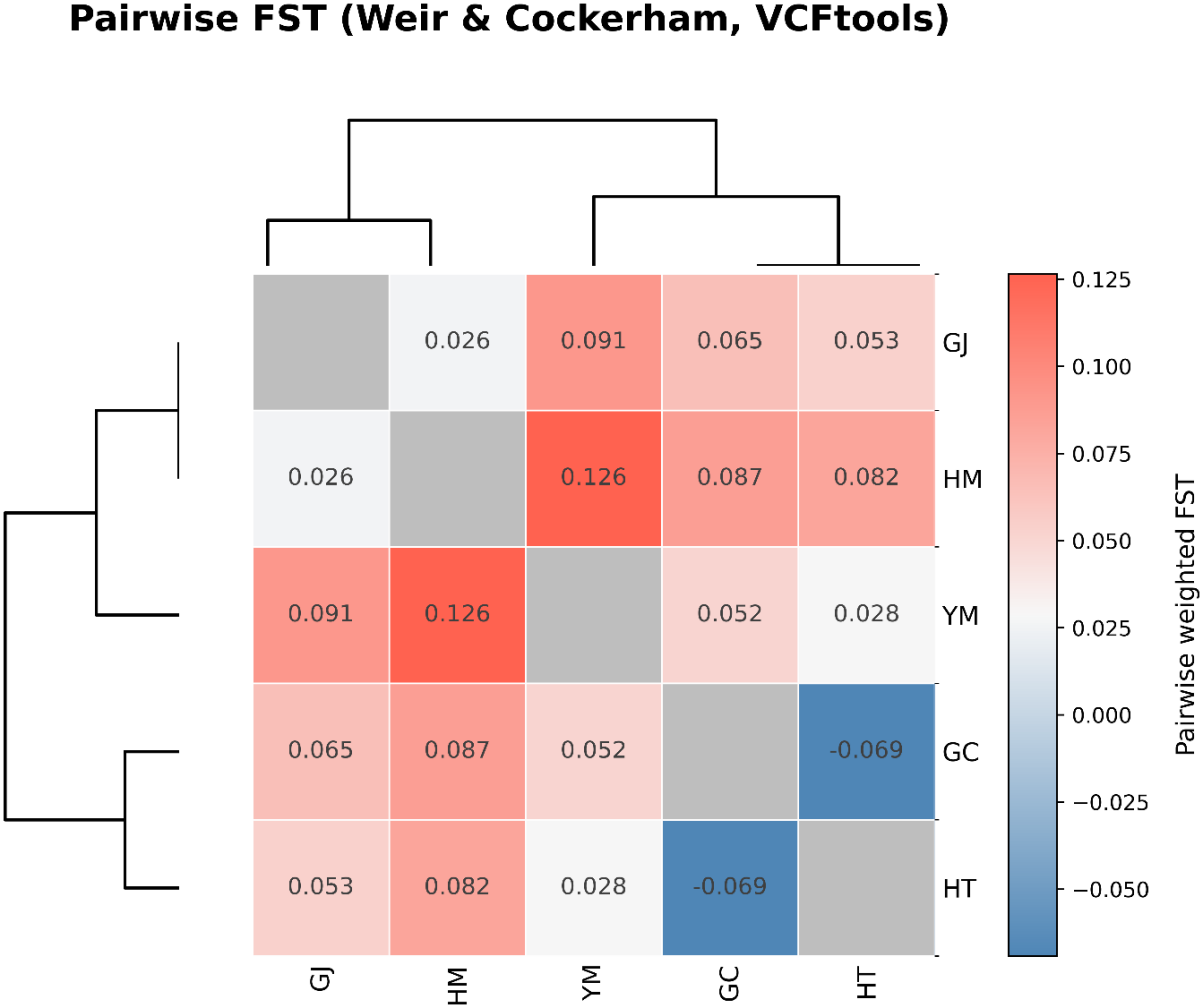
Heatmap of pairwise *F_ST_* values among *Veronica pusanensis* populations. Numbers within squares represent pairwise *F_ST_* estimates.

Pairwise *F_ST_* values showed low to moderate genetic differentiation among populations based on the clone-corrected and LD-pruned 5, 539-SNP dataset (**Figure 4**). The lowest differentiation was observed between GC and HT (*F_ST_* = -0.069), followed by GJ and HM (*F_ST_* = 0.026), indicating close genetic relationships between these population pairs. The highest differentiation was observed between HM and YM (*F_ST_* = 0.126), followed by GJ-YM (*F_ST_* = 0.091), GC-HM (*F_ST_* = 0.087) and HM-HT (*F_ST_* = 0.082). Overall, GJ and HM showed the closest relationship among comparisons involving the wild population, consistent with the PCA and ADMIXTURE results.

To further examine whether the observed genetic differentiation in the wild GJ population may be associated with demographic change, an exploratory demographic analysis was conducted using dadi based on a folded site-frequency spectrum. The two-epoch model showed lower AIC values than the constant-size model across projection settings. However, parameter estimates repeatedly converged near model boundaries, suggesting limited resolution for robust demographic inference (**Supplementary Table 2**).

### 3.6 Effective population size

The effective population size (Ne) differed among populations based on the clone-corrected and LD-pruned 5, 539-SNP dataset (**Table 4**). Contemporary Ne could be estimated for GJ and HM, with point estimates of 260.6 and 95.1, respectively. For GC, HT, and YM, Ne estimates were infinite, indicating that finite estimates could not be obtained from the available clone-corrected samples. These results should therefore be interpreted with caution because of the small number of independent individuals remaining in some populations after clone correction.

**Table 4:** Estimates of effective population size (*Ne*) based on the linkage disequilibrium (LD) method using the clone-corrected and LD-pruned 5, 539-SNP dataset.

Population	Sample size (*N*)	*N*e	95% CI
GJ	20	260.6	238.2-287.6
GC	7	∞	∞-∞
HM	19	95.1	92.7–97.7
HT	5	∞	∞-∞
YM	8	∞	∞-∞

*N*e, effective population size; *N*, sample size; CI, confidence interval.

∞ indicates that no finite Ne estimate could be obtained from the available data.

## 4 Discussion

### 4.1 Genetic diversity of wild *V. pusanensis* compared with other Korean endemic species

Species with narrow geographic distributions and small population sizes generally exhibit lower genetic diversity than widespread taxa (Willi et al., 2006; Li et al., 2012). This pattern is particularly relevant for *V. pusanensis*, a species restricted to the Busan region of Korea (Jang and Noh, 2020), with limited information on its genetic diversity.

The present study revealed that wild *V. pusanensis* (GJ) exhibits moderate genetic diversity (*H*e = 0.293, **Table 3**). This value is comparable to those reported for other Korean endemic plants such as *Phedimus latiovalifolius* (mean *H*e = 0.219), *Sophora koreensis* (mean *H*e = 0.150), and *Forsythia ovata* (mean *H*e = 0.212), all estimated using genotyping-by-sequencing (GBS) approaches (Cho et al., 2024; Ha et al., 2025; Lee et al., 2025). Thus, *V. pusanensis* shows moderate levels of genetic diversity despite the highly restricted distribution.

However, unlike the other taxa, which occur in multiple populations, *V. pusanensis* is known from only a single natural population, making it more vulnerable to genetic drift and environmental change. Moreover, while *F. ovata* and *P. latiovalifolius* generally exhibited low or negative *F_IS_* values across populations, *V. pusanensis* showed a positive *F_IS_* value (*F_IS_* = 0.376), indicating a heterozygote deficit likely associated with inbreeding, spatial genetic structure, or limited effective population size.

### 4.2 Effects of *ex situ* propagation on genetic diversity in *V. pusanensis*

Ex situ–propagated individuals displayed distinct genetic characteristics compared to the wild population of *V. pusanensis*. This pattern is consistent with previous studies reporting increased genetic differentiation between *in situ* and *ex situ* populations (Rucińska and Puchalski, 2011; Lauterbach et al., 2012; Wilson et al., 2017; Forgiarini et al., 2023). *Ex situ* propagation can disrupt gene flow, leading to genetically isolated populations that are more susceptible to genetic drift and altered genetic structure (Li et al., 2018).

In this study, PCA, ADMIXTURE, and pairwise *F_ST_* analyses all revealed genetic differentiation among populations. Notably, HM showed the closest genetic relationship with the wild GJ population. This pattern is consistent with the available propagation record indicating that the HM collection was supplemented in 2018 using seeds derived from individuals from the natural habitat. The use of seed-derived material from the wild population may have helped HM retain genetic variation similar to that of GJ and may explain its close genetic affinity to the wild population. In contrast, HT and YM showed greater genetic differentiation from GJ, and GC showed a mixed or heterogeneous genetic pattern. These patterns suggest that the genetic composition of *ex situ* groups may have been influenced by differences in source material, propagation history, and the number of founder genotypes. However, detailed records on original source individuals, propagation mode, and subsequent propagation history were incomplete for some *ex situ* groups. Therefore, the observed genetic differences among *ex situ* groups cannot be attributed to a single factor.

Furthermore, positive FIS values were observed in all populations, suggesting an overall deficit of heterozygotes that may reflect non-random mating, population structure, or demographic processes. However, the magnitude of FIS varied among populations, and these differences should be interpreted cautiously because clone correction substantially reduced the number of independent individuals in some *ex situ* groups. Finite Ne estimates were obtained only for GJ and HM, whereas the estimates for GC, HT, and YM were infinite. These infinite estimates should not be interpreted as evidence of very large effective population sizes. Rather, they indicate that finite Ne values could not be estimated reliably from the available clone-corrected samples, particularly in populations with small sample sizes. Therefore, the Ne results provide limited support for detailed comparisons among *ex situ* populations.

At the same time, these genetic patterns should be interpreted in light of the limited ecological information currently available for *V. pusanensis*. Although previous studies have provided useful information on seed germination, seedling production, vegetative propagation, and cultivation conditions, species-specific data on pollination biology remain insufficient. Therefore, the genetic differentiation, FIS values, and Ne estimates observed in this study cannot be attributed solely to restricted gene flow, inbreeding, or genetic drift. Instead, they may also reflect differences in *ex situ* propagation history, the number and identity of source individuals, reproductive biology, and demographic processes in the natural habitat. Although the exploratory demographic analysis showed a better fit of the two-epoch model than the constant-size model, parameter estimates were unstable across projection settings (**Supplementary Table 2**). Therefore, additional analyses will be needed to evaluate the demographic history of *V. pusanensis* more reliably.

### 4.3 Conservation implications for *V. pusanensis*

The conservation of *V. pusanensis* requires urgent attention due to its restricted distribution and limited population size. As the species occurs in a single natural population, protecting its habitat must be the highest priority. Any disturbance could have irreversible consequences for its survival. Strict *in situ* conservation measures, including habitat protection and regulation of human activities, are therefore essential (Bracci and Sacchi, 2022; Hoban et al., 2023).

In addition to habitat protection, *ex situ* conservation programs should be carefully designed to support long-term preservation. Because *ex situ* populations may not fully represent the genetic composition of the wild population, propagation strategies should include a sufficiently large and representative number of source individuals to maintain genetic diversity and reduce the risk of further genetic imbalance (Frankham et al., 2011; Mounce et al., 2017; Forgiarini et al., 2023). The close genetic similarity between HM and the wild GJ population suggests that HM may represent an important *ex situ* resource for conservation, particularly because this group was supplemented in 2018 using seed-derived material from the natural habitat.

The clone-correction analysis showed that some *ex situ* groups contained many genetically redundant individuals, indicating that the number of maintained plants may not reflect the number of independent genotypes. Therefore, *ex situ* management should focus not only on increasing the number of individuals but also on maintaining genetically distinct lineages. Periodic supplementation with genetically diverse individuals or seed-derived material from the wild population may help maintain genetic representation over time, provided that collection does not adversely affect the natural population.

Maintaining connectivity among managed populations may also support genetic stability. Controlled exchange of individuals or genetic material could be beneficial, but such actions must be implemented cautiously to avoid unintended genetic or ecological consequences. The finite Ne estimates obtained for GJ and HM may provide useful demographic information, whereas the infinite estimates for GC, HT, and YM indicate that reliable finite values could not be obtained from the available clone-corrected samples. Therefore, these estimates should not be used to rank the genetic vulnerability of *ex situ* populations.

For populations with critically low viability, reinforcement through reintroduction or supplementation may be beneficial (Houde et al., 2015). However, any reinforcement should be implemented only after evaluating genetic compatibility among potential source populations. The use of genetically similar and well-documented source materials would be necessary to reduce the risk of outbreeding depression and to maintain locally adapted genetic variation (Reynolds et al., 2012; Hohenlohe et al., 2021; Wei et al., 2023). In addition, information on propagation history, maternal lineages, source-population identity, and clone-group membership should be incorporated into future *ex situ* and restoration programs.

Overall, integrating genomic information with ecological and propagation data will be essential for the effective conservation of *V. pusanensis*. Continued monitoring, improved documentation of *ex situ* collections, periodic evaluation of genetic redundancy, and future studies on pollination, seed dispersal, recruitment, and habitat conditions will further support the development of adaptive and sustainable conservation strategies.

## Data Availability

The datasets presented in this study can be found in online repositories. The names of the repository/repositories and accession number(s) can be found below: https://www.ncbi.nlm.nih.gov/, PRJNA1455860.
