# Supplementary material for: Restricted distribution and population genetic structure in the rare Korean endemic plant Veronica pusanensis: implications for conservation
Source: Front Plant Sci. 2026 Jul 29;17:1861445. doi: 10.3389/fpls.2026.1861445 (PMC13462074; doi:10.3389/fpls.2026.1861445)
Supplement: Supplementary file 1 [file DataSheet2.docx]

**Supplementary Fig S1.** Sensitivity of clone identification to pairwise multilocus genotype similarity thresholds. A: Changes in the number of individuals assigned to putative clonal groups, individuals removed after clone correction, and inferred clonal groups across similarity thresholds ranging from 80.0% to 99.5%. B: Number of individuals removed from each population across the same threshold range. The dashed vertical line indicates the selected threshold of 94%.

**Supplementary Fig S2.** GenomeScope profile based on the 21-mer frequency distribution of *Veronica pusanensis*.

**Supplementary Fig S3.** Principal component analysis based on PC3 and PC4 using the clone-corrected LD-pruned 5,539-SNP dataset. PC3 and PC4 explained 6.89% and 5.88% of the total genetic variation, respectively.
